# Clinical performance of posterior resin composite restorations bonded with universal adhesive system using three different application modes: a 26-month randomized clinical trial

**DOI:** 10.1590/1678-7757-2025-0402

**Published:** 2025-11-17

**Authors:** Thaís Silva MENDONÇA, Andreia Assis CARVALHO, Jéssica Karla Maia ZAGO, Terezinha de Jesus Esteves BARATA, Érica Miranda TORRES, Gersinei Carlos de FREITAS, Lawrence Gonzaga LOPES

**Affiliations:** 1 Universidade Federal de Goiás Faculdade de Odontologia Departamento de Reabilitação Oral e Prevenção Goiânia GO Brasil Universidade Federal de Goiás, Faculdade de Odontologia, Departamento de Reabilitação Oral e Prevenção, Goiânia, GO, Brasil.

**Keywords:** Universal adhesives, Randomized clinical trial, Dental restorations, Clinical longevity

## Abstract

**Objective:**

This study aimed to evaluate the clinical performance of posterior resin composite restorations (Class I and II), bonded with a universal adhesive using three application modes over 26 months, comparing the adapted FDI and USPHS criteria.

**Methodology:**

In total, 138 Class I and II cavities were restored using Scotchbond Universal adhesive (3M ESPE) in three application modes: etch-and-rinse (ER); selective enamel etch (SEE), and self-etch (SE). All cavities were restored with FiltekTM Supreme composite resin (3M ESPE). The restorations were evaluated by two trained and calibrated evaluators using FDI and adapted USPHS criteria at baseline (12.02±5.68 days) and after 26 (± 2.36) months. The obtained data were subjected to statistical analysis between the groups using the Friedman and Wilcoxon test for intra-group analysis and the McNemar test for comparisons between FDI and USPHS criteria (α=0.05).

**Results:**

No statistically significant differences were observed between the ER, SEE, and SE groups after 26 months, except for marginal discoloration between the ER and SE groups (p≤0.05).

**Conclusion:**

Class I and II resin composite restorations performed with Scotchbond Universal adhesive using ER, SEE, and SE modes showed acceptable clinical performance over a 26-month evaluation period. Moreover, the adapted FDI and adapted USPHS criteria yielded comparable results. Brazilian Clinical Trials Registry (Rebec) — RBR-9p3hdp

## Graphical abstract

**Figure:**
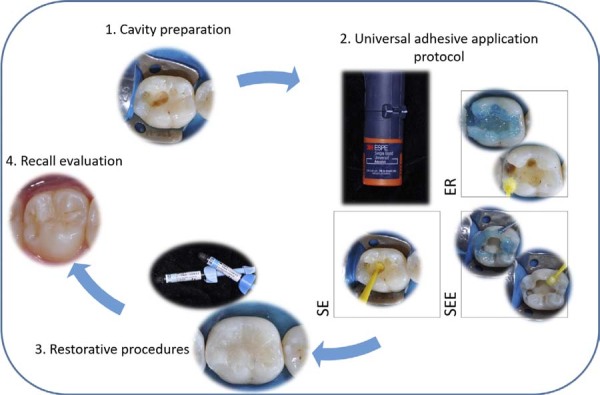

## Introduction

Over time, adhesive systems have become integral to dental practice; yet they continue to face challenges regarding the durability of the resin-dentin interface. To address these issues, significant modifications have been made to the composition and application methods of these systems to enhance clinical efficacy and streamline procedures for direct and indirect restorations. Consequently, a generation of adhesive systems, known as “universal” or “multi-mode” adhesives, has emerged.^1^ These innovative systems offer versatile solutions, providing dental professionals with improved performance and adaptability across various clinical scenarios.^2,3^

These adhesives represent the most advanced offerings currently available on the market.^4,5^ Their enhanced versatility is primarily attributed to the incorporation of functional monomers, particularly 10-methacryloyloxydecyl dihydrogen phosphate (10-MDP).^6^ This monomer is a key component of universal adhesives, renowned for its ability to form strong and stable chemical bonds with the hydroxyapatite in enamel and dentin.^6,7^ The strong chemical bonds established by 10-MDP significantly contribute to the creation of a stable adhesive interface, thus enabling different application protocols.^7^

Among the application modes for universal adhesives, they can be employed in the traditional etch-and-rinse (ER) mode (which involves conditioning both enamel and dentin), selective enamel etch (SEE) mode, or in the self-etch (SE) mode without prior conditioning of the dental substrate. Additionally, these adhesives can be used on dry or moist dentin surfaces following conditioning with phosphoric acid.^8^ A study has evaluated the performance of cervical restorations post-application 18 and 36-months that used ER, SEE, and SE conditioning techniques indicated no significant differences in retention rates between the three methods at both evaluation periods, suggesting that universal adhesives maintain effective bonding performance regardless of the conditioning technique.^8-12^ However, no study has evaluated restorations in Class II posterior teeth.

Studies have indicated that ER shows superior clinical performance: higher retention rates and reduced marginal discoloration.^1,12,13^Specifically, a study on Class V restorations has reported that the technique described by Perdigão and Lougercio^1^ (2014) failed to significantly enhance the retention rate of universal adhesives when compared to application on dry dentin.

Nevertheless, the literature has a notable absence of published long-term clinical studies spanning over 26 months that conclusively show whether ER consistently outperforms SE when universal adhesives are used in Class I and II posterior restorations. Therefore, comprehensive long-term clinical trials are essential to evaluate how different strategies involving universal adhesives may optimize clinical outcomes.

Over the years, few studies have investigated the clinical efficacy of these materials in Class I and II restorations.^14-17^ Within this context, evaluation of restorations involves criteria considered relevant to the clinical performance of dental restorative materials. Evaluations can be judged according to various factors to assess the clinical performance of the restorative material. Such assessments may involve various factors that can assess the clinical efficacy of restorative materials across clinical outcomes.^18,19^ Therefore, this randomized double-blind clinical trial aimed to study the influence of different application strategies on the clinical behavior of a universal adhesive over the course of 26 months in posterior dental restoration (Class I and II) using two evaluation criteria; those of the World Dental Federation (FDI), adapted United States Public Health Service (USPHS).

## Methodology

### Study design

This randomized, double-blind, split-mouth clinical trial began in June 2015 and ended in April 2018. The CONSORT guidelines were followed.^20^ All interventions were conducted at the dental clinic of the Faculty of Dentistry, Federal University of Goiás.^5^

The research project was approved by the Research Ethics Committee (CAAE: 36829814.0.0000.5083). This clinical trial was registered in the Brazilian Clinical Trials Registry (Rebec)—a national system of Clinical Trials Registry—under Protocol RBR-9p3hdp, on May 24, 2015.

### Sample size

The sample size was 50 teeth per group based on rates of marginal discoloration, postoperative sensitivity, adaptation, and retention of Scotchbond Universal adhesive (3M ESPE, St Paul, MN, USA) in Class V restorations after 18 months.^9^The choice of a study with Class V restorations is due to the absence of published investigations on universal adhesives in Class I and II cavity preparations.

### Eligibility criteria

The following were used as inclusion criteria: age 18 years or older, clinical and radiographic need for Class I and/or II restorations in permanent posterior teeth due to caries lesions and/or unsatisfactory pre-existing restorations, presence of at least three teeth (one for each adhesive protocol) to be restored per participant with present antagonist, and at least 20 teeth in functional occlusion.

The following were used as exclusion criteria: periodontitis, users of orthodontic or removable appliances, users of removable prostheses, signs of parafunctional habits, pregnant or lactating women, and enamel fluorosis.

To assist in diagnosis, digital periapical and interproximal radiographic examinations were used for participants who met the inclusion criteria during the clinical examination. Moreover, participants received oral hygiene instructions prior to restorative procedures.

### Randomization and blinding

Subjects were randomized using manual tables following the order in which they were examined. The teeth requiring restoration were numbered according to the universal system for premolars and molars regardless of the class of the restorations. The three groups were allocated according to the universal adhesive application protocol: ER, SEE, and SE, varying the restoration sequence from 1 to 3. Each participant had three teeth to be restored; a separate draw was performed for each tooth: the first tooth received ER, SEE, and SE.

Participants were blinded to allocation as they were unaware of which protocol was applied to each tooth. Randomization was performed by a researcher who participated in no clinical procedure. Information regarding group and sequence was recorded on sealed envelope forms, and only at the time of the procedure the operator had access to the assigned protocol. Both patients and examiners remained blinded to the adopted protocol.

### Restorative procedures resin

**Figure 1:**
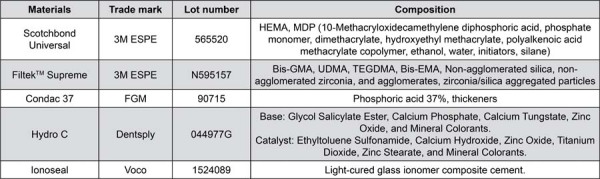
Composition of the main materials used in this study as informed by the manufacturers. Abbreviations: Bis-GMA - bisphenol A diglycidyl ether dimethacrylate; Bis-EMA - bisphenol A polyethylene glycol diether dimethacrylate; HEMA - 2-hydroxyethyl methacrylate; TEGDMA - triethylene glycol dimethacrylate; UDMA - diurethane dimethacrylate.5

Clinical procedures were performed by two operators who are specialists in restorative dentistry. One of them was responsible for the operative procedures, whereas the other, for restorative procedures. The materials in this study are described in Figure 1.

After application of local anesthesia and absolute isolation, the carious lesion or unsatisfactory restoration was accessed with spherical diamond burs (KG Sorensen, Cotia, SP, Brazil) with a number compatible with the carious lesion or replacement of the unsatisfactory restoration at high speed under refrigeration (Kavo, Rio de Janeiro, RJ, Brazil). Then, the carious dentin was removed with spherical carbide steel burs (Maillefer, Dentsply, Rio de Janeiro, RJ, Brazil) at low speed (Contra-angle, Kavo, Rio de Janeiro, RJ, Brazil).

The depth of the caries was estimated by an interproximal radiographic examination at the initial diagnosis (visually confirmed with a millimeter probe). For cavities considered very deep, participants’ pulp was protected using calcium hydroxide cement (Hydro C, Dentsply Sirona, North Carolina, USA). Thereafter, resin-modified glass ionomer cement (Ionoseal, Voco, Cuxhaven, Germany) was applied and light-cured for 20 seconds with a LED curing device (Ultraled, Dabi Atlante, Ribeirão Preto, SP, Brazil). In deep cavities, only resin-modified glass ionomer cement was used. In moderate or shallow cavities, the adhesive protocols were applied directly.

The tooth surface was treated with the Scotchbond Universal adhesive system (3M ESPE) in adherence to established protocols. For ER group (control group), the procedure followed the manufacturer’s recommendations, utilizing 37% phosphoric acid (Condac 37, FGM, Joinville, SC, Brazil) for 30 seconds on enamel and 15 seconds on dentin. Subsequently, the surface was thoroughly rinsed with a water jet for 10 seconds, and excess moisture was eliminated using absorbent paper. The adhesive was actively applied to the tooth surface using an applicator tip for 20 seconds, followed by a 5-second air jet, adhering strictly to the manufacturer’s guidelines. Final curing was carried out for 20 seconds using the LED curing device, with an irradiance of 500 (±50) mW/cm^2^. The device was connected to a power stabilizer and the power density was verified in advance using a radiometer (Demetron Research Corp.), using a power of 600 mW/cm^2^.

In the second protocol, SEE was implemented, involving the application of 37% phosphoric acid exclusively on enamel for 30 seconds, followed by a thorough water jet for 10 seconds. Excess moisture was removed using absorbent paper, and the adhesive was applied as per the previous group’s procedure.

In the SE group, cavities were dried with absorbent paper. Subsequently, the adhesive was actively applied to the tooth surface using an applicator tip for 20 seconds, followed by a 5-second air jet, without any preliminary dentin surface preparation.

After application and polymerization of the adhesive, Filtek™ Supreme composite resin (3M ESPE) was inserted in 2-mm oblique increments and light-cured for 20 seconds per increment, followed by a final polymerization period of 40 seconds using the same curing light. For Class II restorations, a sectional metal matrix, available in three sizes (Unimatrix, TDV Dental, Pomerode, SC, Brazil), were used with wooden wedges. After occlusal adjustments, the restorations were finished with fine diamond burs (KG Sorensen, Serra, ES, Brazil) and/or scalpel blades (Lamedid Comercial e Serviços Ltda, Barueri, SP, Brazil) and polished with siliconized rubber burs (FlexiCups and FlexiPoints, Cosmedent, Chicago, IL, USA) and a felt disc (Diamond, FGM, Joinville, SC, Brazil) coated with diamond paste (Diamond Excel, FGM).

### Recall evaluation

**Figure 2:**
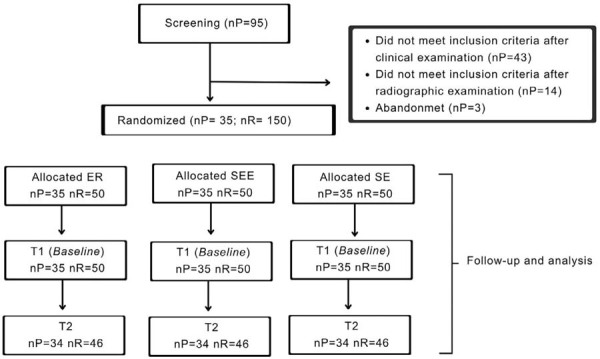
Characteristics of the participants of this study (nP– number of participants; nR – number of restorations; ER - etch-and-rinse; SEE – selective enamel etch; SE – self-etch; T1 – baseline; T2 - 26.28 (± 2.36) months.

The first evaluation (T1) took place from 7 to 21 days (mean 12.02±5.68 days) following the restoration procedure, whereas the second one (T2) occurred from 22 to 30 months on average (mean 26.28±2.36 months) (Figure 2). Before each evaluation, dental prophylaxis was performed using a pumice stone and water suspension.

Subsequently, evaluations were conducted using an air jet followed by visual inspection with a clinical mirror, explorer probe, and dental floss for assessing proximal surfaces, in accordance with the methodology outlined by Hickel, et al.^18^ (2023). The evaluations were conducted by two trained examiners who were not involved in the restorative procedures (thus blinded to group assignment). Both examiners independently evaluated the restorations at a single time point. Prior to the evaluation, the evaluators underwent calibration training using 40 clinical images that represented the scoring system (Kappa >0.80).^18^

**Figure 3:**
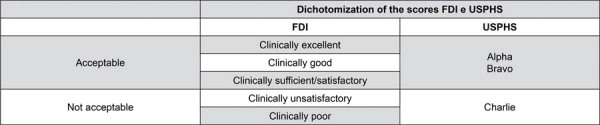
Dichotomization of FDI and USPHS scores to enable comparisons.

**Figure 4:**
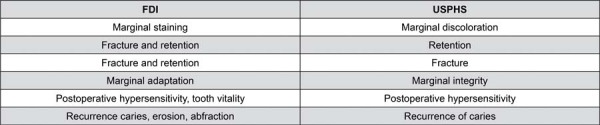
Comparison between criteria analyzed: FDI and USPHS.

Restorations were to be evaluated based on FDI criteria for aesthetic, functional, and biological properties,^18^ as well as adapted *Alpha, Bravo*, and *Charlie* criteria from adapted USPHS.^19^ The FDI and USPHS criteria were compared and scores were dichotomized into “acceptable” and “not acceptable” (Figure 3). Specific properties assessed by each criterion were separately analyzed (Figure 4): FDI and adapted USPHS. In cases of disagreement during evaluations, examiners reached a consensus on the final classification criteria for the evaluated tooth prior to dismissing the patient.

### Data analysis

Descriptive analysis was performed for the evaluated criteria. Considering that the data were paired, the Friedman test (α=0.05) was used to compare the groups at both times (T1 and T2). In cases of statistically significant differences, the Wilcoxon test with Bonferroni correction (α=0.01) was used. The Wilcoxon test (α=0.05) was used to compare T1 and T2 times for each group, whereas the McNemar test was used (α=0.05) to compare the FDI and USPHS criteria.

## Results

In total, 138 restorations were evaluated. The data pertaining to participants and the types of restorations evaluated are included. All baseline details relative to the research subjects and characteristics of the restored are shown in Table 1.

**Table 1:** Distribution of participants and restored teeth.

Groups	Gender		Age (years)		Tooth		Arcade distribution		Type of preparation		Cavity Depth			
	M	F	20-29	30-50	PM	M	Max	Mand	I	II	S	M	D	VD
ER	14	21	27	8	10	40	33	17	34	16	15	4	16	15
SEE					8	42	29	21	30	20	19	1	18	12
SE					11	39	26	24	32	18	26	6	11	7
A Freq	14	21	27	8	29	121	88	62	96	54	60	11	45	34
R Freq	40%	60%	77%	23%	19%	81%	58%	42%	64%	36%	40%	7%	30%	23%

Abbreviations: ER- etch-and-rinse; SEE- selective enamel etch; SE- self-etch; A Freq - Absolute frequency; R Freq - relative frequency; M- male; F- female; PM- premolar; M- molar; Max- maxilla; Mand- mandible; I- Class I; II- Class II; S- shallow; M- moderate; D- deep; VD- very deep.

According to the FDI and adapted USPHS criteria, the restorations were evaluated from 7 to 21 (baseline, 12.02±5.68) days after the restoration (T1, n=50 per group) and the second assessment, from 22 to 30 (T2, 26.28±2.36) months (n=46 per group). The findings of the evaluation are detailed in Table 2 (USPHS) and Table 3 (FDI). Restorations were classified as “acceptable” and “not acceptable.” When the groups (ER, SEE, and SE) were compared at the same time, no criteria showed statistical differences between groups.

**Table 2:** Number of evaluated restorations for each experimental group, classified according to the adapted USPHS criteria at T1 and T2 times.

		Decision	T1			T2		
			ER	SEE	SE	ER	SEE	SE
Marginal staining	Alpha	Acceptable	50	50	50	37	44	37
	Bravo		-	-	-	9	2	9
	Charlie	No acceptable	-	-	-	-	-	-
Retention	Alpha	Acceptable	50	49	50	46	46	45
	Bravo		-	-	-	-	-	1
	Charlie	No acceptable	-	1	-	-	-	-
Fracture	Alpha	Acceptable	50	50	50	45	46	46
	Bravo		-	-	-	1	-	-
	Charlie	No acceptable	-	-	-	-	-	-
Marginal adaptation	Alpha	Acceptable	50	50	49	46	45	45
	Bravo		–	-	1	–	1	1
	Charlie	No acceptable	-	-	-	-	-	-
Postoperative sensitivity	Alpha	Acceptable	47	47	47	46	46	46
	Bravo		-	-	-	-	-	-
	Charlie	No acceptable	3	3	3	-	-	-
Recurrence of caries	Alpha	Acceptable	50	50	50	46	46	46
	Bravo		-	-	-	-	-	-
	Charlie	No acceptable	-	-	-	-	-	-

Abbreviations: T1- Baseline; T2 - 26.28 (± 2.36) months; ER - etch-and-rinse; SEE - selective enamel etch; SE - self-etch.

**Table 3:** Number of evaluated restorations for each experimental group, classified according to the FDI criteria at T1 and T2 times.

Esthetic Properties	Escore	Decision	T1			T2		
			ER	SEE	SE	ER	SEE	SE
Staining margin	1	Acceptable	50	50	49	37	44	37
	2		-	-	1	9	2	9
	3		-	-	-	-	-	-
	4	No acceptable	-	-	-	-	-	-
	5		-	-	-	-	-	-
**Functional Properties**			**T1**			**T2**		
			**ER**	**SEE**	**SE**	**ER**	**SEE**	**SE**
Fracture of material and retention	1	Acceptable	50	49	50	45	46	45
	2		-	1	-	-	-	1
	3		-	-	-	1	-	-
	4	No acceptable	-	-	-	-	-	-
	5		-	-	-	-	-	-
Marginal adaptation	1	Acceptable	50	50	49	46	45	45
	2		-	-	1	–	1	1
	3		-	-	-	-	-	-
	4	No acceptable	-	-	-	-	-	-
	5		-	-	-	-	-	-
**Biological Properties**			**T1**			**T2**		
			**ER**	**SEE**	**ER**	**SEE**	**ER**	**SEE**
Prostoperative (hyper-) sensitivity and tooh vitality	1	Acceptable	47	47	47	46	46	46
	2		3	3	3	-	-	-
	3		-	-	-	-	-	-
	4	No acceptable	-	-	-	-	-	-
	5		-	-	-	-	-	-
Recurrence of caries (CAR) erosion, abfraction	1	Acceptable	50	50	50	46	46	46
	2		-	-	-	-	-	-
	3		-	-	-	-	-	-
	4	No acceptable	-	-	-	-	-	-
	5		-	-	-	-	-	-

Abbreviations: T1- Baseline; T2 - 26.28 (± 2.36) months; ER - etch-and-rinse; SEE - selective enamel etch; SE - self-etch.

### Esthetic properties

Compared with baseline, all groups showed Bravo scores for marginal staining at T2: ER n=9 with, *Bravo* score (p=0.001); SEE n=2, *Bravo* score (p=0.023), and SE n=9, *Bravo* score (p=0.001). Restorations showed satisfactory outcomes, receiving a “*Bravo*” rating according to the USPHS criteria and a classification of “clinically good” according to the FDI criteria in both groups.

### Functional properties

The restorations were initially evaluated as clinically excellent based on the FDI criteria. However, at the 26-month evaluation, 138 restorations were assessed as marginally acceptable according to both the FDI and USPHS criteria. This shift highlights the importance of long-term follow-up in assessing the durability and clinical performance of dental restorations. Although no significant differences were observed in the retention rate initially, at the 26-month follow-up, statistically significant differences emerged between the ER and SE groups (p=0.034 for ER and p=0.034 for SE).

### Biologics properties

At baseline, nine restorations showed postoperative sensitivity. However, no sensitivity was recorded in any restoration at 26.28 (±2.36) months. A statistically significant reduction in postoperative sensitivity was observed from baseline to T2. No cases of secondary caries were detected in any restoration at T2. According to the statistical analysis, no significant differences were found between adhesive application modes (p>0.15).

Statistical analysis found no significant difference when FDI criteria and adapted USPHS were compared (p≥ .05).

## Discussion

In this study, the clinical behavior of Class I and II composite resin restorations using the three modes of application of the universal adhesive (ER, SEE, and SE) was evaluated over a period of 26 (± 2.36) months. The results of this study indicated no significant differences in clinical performance between the modes of application of the universal adhesive.

The researchers used the FDI and adapted USPHS evaluation criteria (which are commonly used in clinical trials) to evaluate dental restorations. These criteria are important to find the properties and performance of dental materials. The FDI criteria offer a broader approach to evaluating the clinical performance of materials when compared to the adapted USPHS criteria.^18,19^ The FDI framework evaluates more comprehensive aspects and characteristics of dental materials, providing more detailed assessments.^18^ Studies show that both are effective for obtaining information but may show differences and specificities in the evaluation of certain parameters. Despite their differences, both these important criteria were used in the evaluations in this study to ensure a robust assessment.^9,13^

At follow-up, the ER and SE groups showed a higher number of restorations classified as score 2 (FDI) and Bravo (USPHS) for marginal staining when compared with the SEE group, despite statistically insignificant differences. Over the evaluation periods, the restorations showed statistically significant changes in marginal discoloration, indicating temporal variation in performance between groups. Nevertheless, after 26 months, this clinical outcome remained within acceptable parameters. Comparable findings have been reported in other longitudinal clinical investigations.^9,15,21-26^ These ratings indicate that the restorations sustained consistent and adequate performance throughout the evaluation period. The “*Bravo*” rating from USPHS evinces that the restorations maintained an acceptable standard in marginal staining and other clinical parameters, whereas the “clinically good” designation from FDI reflects that the restorations met satisfactory aesthetic standards.

No failures were observed in the marginal adaptation and retention of the restorations. Therefore, the type of adhesive system applied failed to influence the longevity of the restorations over a 26-month clinical follow-up period. The chemical bonding potential of 10-MDP is fundamental to establishing a durable and stable adhesive interface, enabling clinicians to customize bonding strategies to the specific demands of each clinical scenario and optimize clinical outcomes.^6,7,27^ Complementing this effect, 2-hydroxyethyl methacrylate, and its pronounced hydrophilicity, enhances substrate wetting and promotes higher bond strengths.^28^ When combined with Bis-GMA, these monomers synergistically reinforce the adhesive interface, further improving the overall performance of the bonding system.^6,29^

Regarding the occurrence of caries lesions at the margins of the restorations, the participants in this study showed good general health and acceptable oral hygiene, and no group showed the incidence of carious lesions, a recurring finding in other clinical trials.^5,13,24,30^ However, a systematic analysis of caries risk assessment in participants would be crucial to understand its impact on the clinical performance of the restoration.^22,30,31^ This is particularly relevant because restorations in patients with a high caries risk may have higher failure rates than those in low-risk patients. Moreover, patient-related factors—particularly caries risk, as well as the size, depth, and location of the restoration—may more greatly influence restoration longevity than the adhesive strategy itself,^33^ underscoring the importance of incorporating risk-based stratification into future clinical trials.

Regarding post-operative sensitivity, no differences were observed between the groups, irrespective of the adhesive technique. Similarly, in studies^24,32^ evaluating the SE mode, no sensitivity was reported at any follow-up time. In a study with a mean follow-up of 12 (±2.7) months, no statistically significant differences were detected between groups, and no association was found between postoperative sensitivity and cavity depth.^5^ The same results were found in other studies that used system application modes.^9,26^ Systematic reviews with meta-analyses suggested that universal adhesives are associated with a lower incidence of hypersensitivity.^4,31^

In this study, it is important to note that all restorations were carried out under the same conditions by the same operator in all cavities. In other studies, restorations were carried out by more than one operator, which may have impacted results. The longevity of restorations depends on many factors; the extent of cavity size is a critical factor that can influence the survival of posterior restorations (Class I or II).^14,33^ Studies have shown that, regardless of material, restorations with “large cavities” have higher failure rates than those with “small cavities”.^14^ Most studies show that the ER technique is superior to the self-etching technique for some clinical outcomes,^34-36^ whereas in other studies,^3,23,36,37^ no differences were observed between modes regarding the longevity of composite resin restorations (the same result obtained in this study).

Previous systematic reviews and meta-analyses have reported that the clinical performance of universal adhesives is generally satisfactory in the short and medium term, with no consistent differences between application strategies, although long-term studies (>5 years) are still required to confirm these trends.

In summary, this 26-month randomized clinical trial showed that Class I and II posterior composite restorations bonded using a universal adhesive system showed satisfactory clinical performance. Importantly, the mode of adhesive application - ER, SEE, or SE – failed to significantly affect clinical outcomes. These findings indicate that clinicians can tailor the adhesive strategy to case-specific requirements without compromising the medium-term success of restorations. Failures in posterior teeth can be observed in periods exceeding of follow-up.^10,12,14,33^ Longer and multicenter clinical trials remain essential to validate the equivalence of application strategies and to assess the durability of universal adhesives in posterior teeth over extended periods.

## Conclusions

Class I and II resin composite restorations performed with Scotchbond Universal adhesive using ER, SEE, and SE modes showed acceptable clinical performance over a 26-month evaluation period. Moreover, the adapted FDI and adapted USPHS criteria yielded comparable results.
